# Sequel and Ultimate Result of a Case of Resection of the Knee Joint

**Published:** 1859-06

**Authors:** Daniel Brainard

**Affiliations:** Surgeon of the U. S. Marine Hospital at Chicago, Etc.


ARTICLE IV.

### SEQUEL AND ULTIMATE RESULT OF A CASE OF RESECTION OF THE KNEE JOINT.

BY DANIEL BRAINARD, M. D, SURGEON OF THE U. S. MARINE HOSPITAL AT CHICAGO, ETC.

In the number of this Journal for July, 1858, I reported a case of resection of the lower end of the femur for scrofulous disease, in a boy ten years old. This operation was performed January 20th, 1858, and the case was reported as successful.

In the discussion to which that case gave rise, in the Society of Surgery of Paris (see report in the May number of this journal), it was objected, that the ultimate result and state of the facts were not, and, at that period, could not, be ascertained. This objection seemed perfectly well founded, and I, therefore, took occasion to re-examine this subject, which was done May 13th, 1859, in presence of Drs. Powell and Durham, and the pupils employed at the Marine Hospital in this city. The result was as follows:

1. The incisions are all perfectly healed, and the knee is free from pain and tenderness.

2. Bony union has perfectly taken place; but this seems to have been complete only seven or eight months after the operation.

3. The member, measuring from the pelvis to the ankle, is two and a half inches shorter than the other. This is the exact length of the piece removed, so that the member must either have grown as fast as the other for about sixteen months, or the space between the thigh and femur was filled up with bony substance.

4. The boy steps and walks with the member without any pain or difficulty, except what results from the shortness. With a high-heeled shoe, he could walk well.

I do not think it admits of a question, that this member is at the present time, much more useful than any artificial member could be.

